# Photodegradable by Yellow-Orange Light degFusionRed Optogenetic Module with Autocatalytically Formed Chromophore

**DOI:** 10.3390/ijms24076526

**Published:** 2023-03-30

**Authors:** Konstantin G. Chernov, Kyrylo Yu. Manoilov, Olena S. Oliinyk, Daria M. Shcherbakova, Vladislav V. Verkhusha

**Affiliations:** 1 Medicum, Faculty of Medicine, University of Helsinki, 00290 Helsinki, Finland; 2 Department of Genetics and Gruss-Lipper Biophotonics Center, Albert Einstein College of Medicine, Bronx, NY 10461, USA

**Keywords:** degron, induced degradation, SuperNova, KillerRed, miniSOG, spectral multiplexing

## Abstract

Optogenetic systems driven by yellow-orange light are required for the simultaneous regulation of several cellular processes. We have engineered the red fluorescent protein FusionRed into a 26 kDa monomeric optogenetic module, called degFusionRed. Unlike other fluorescent protein-based optogenetic domains, which exhibit light-induced self-inactivation by generating reactive oxygen species, degFusionRed undergoes proteasomal degradation upon illumination with 567 nm light. Similarly to the parent protein, degFusionRed has minimal absorbance at 450 nm and above 650 nm, making it spectrally compatible with blue and near-infrared-light-controlled optogenetic tools. The autocatalytically formed chromophore provides degFusionRed with an additional advantage over most optogenetic tools that require the binding of the exogenous chromophores, the amount of which varies in different cells. The degFusionRed efficiently performed in the engineered light-controlled transcription factor and in the targeted photodegradation of the protein of interest, demonstrating its versatility as the optogenetic module of choice for spectral multiplexed interrogation of various cellular processes.

## 1. Introduction

A variety of optogenetics tools (OTs) have been developed for the temporal and spatial light regulation of biological processes in cells and organisms. Most of the biochemical pathways are regulated in several control points, and a combination of multiple OTs regulated by distinct wavelengths of light [1,2,3] is required for effective control of metabolic and signaling pathways, as well as complex physiological processes [4,5]. However, current OTs are limited by spectral ranges that activate their photosensory modules.

Several types of photosensory domains used to engineer OTs are available. A UVR8 domain that binds its partner COP1 [1] requires UV light (280–315 nm) for this. The photocleavage of protein PhoCl [6] is induced by violet light (~380 nm). Blue light regulates several types of photosensors. One type incorporates FMN as a chromophore: light-oxygen-voltage domain 2 (LOV2) [7], GIGANTEA [8], Vivid domain (VVD) [9] and its derivatives, including Magnets [10] and TULIPS [11] (VVD and its derivatives incorporate both FMN and FAD). The second type incorporates only FAD as a chromophore: cryptochrome 2 (CRY2) [12] and BLUF domain [13]. The third type incorporates p-coumaric acid as a chromophore: photoactivatable yellow protein (PYP) [7]. Cyan light (~500 nm) induces the dissociation of tetrameric Dronpa [14] and dimeric pdDronpa [15] proteins. Green light activates bacterial transcription factor CarH (~525 nm), requiring cyanocobalamin (vitamin B12) [16] and the dissociation of PCB-dependent cyanobacteriochrome Am1_c0023g2 (~525 nm) and its partner BAm green [17]. Far-red (650–750) light activates the binding of PCB-dependent phytochrome B (PhyB) to PIF3 [18] or PIF6 [19]; biliverdin-dependent photosensors include c-di-GMP-producing BphS (~730 nm) [20], MagRed pair [21] (~660 nm) and oligomerizing iLight [22] (~660 nm). Near-infrared (NIR) light (750–900 nm) activates biliverdin-dependent bacteriophytochrome-based *Rp*BphP1-PpsR2 [23] and *Rp*BphP1-QPAS1 [2] (~780 nm) pairs. However, currently, there are no OTs regulated by a yellow-orange (550–600 nm) spectral range.

Despite the large success of OT applications, there are several important limitations. For example, UV light causes DNA damage, such as cyclobutane-pyrimidine dimers (CPDs) and 6-4 photoproducts (6-4PPs), leading to double-strand breaks and apoptosis induction [24]. Violet and blue light also exhibit notable cytotoxicity [25]. Additionally, various cofactors that serve as chromophores for photosensory domains may be produced in insufficient quantities or be absent in mammalian cells (for example, PCB). Moreover, some photosensors, such as CRY2 and *Rp*BphP1, are large (MW of 80–100 kDa) and possess a multidomain structure. Because of this, some OTs engineered from them cannot be packed into adeno-associated virus (AAV) particles, thus limiting their DNA delivery in vivo.

In this regard, photosensory OT modules based on fluorescent proteins (FPs) of a GFP-like family or proteins, such as Dronpa [14] and pdDronpa [15], have important advantages, including small size (~26 kDa) and autocatalytic formation of the chromophore via the cyclization of their amino acid residues. Although the exact mechanism of photoinduced dissociation of Dronpa and its derivatives is unknown, available data suggest that photoinduced *cis-trans* chromophore isomerization leads to a notable rearrangement of amino acid residues in the conservative A/C dimerizing interface, which consists of β-sheets 7 and 8 of the protein β-can of the GFP-like proteins [26,27]. Since Dronpa variants are regulated by cyan light, engineering FP-based OTs sensing yellow-red light is desirable.

The regulation of OTs in cells can be carried out in various ways. Most often, OTs can only be turned “ON” and “OFF” while their presence in cells is permanent. The regulation of the level of these OTs may require additional genetic systems for transcriptional OT control or directing excess OT molecules to proteasomes [28]. Another OT mode of action is a chromophore-assisted light inactivation (CALI) [29] approach, consisting of the generation of reactive oxygen species (ROS), which inactivate proteins of interest (POIs) located nearby or fused to OT. Representative examples of these OTs are KillerRed [30], SuperNova [29] and miniSOG [31]. However, the generation of ROS in cells may negatively affect their physiology.

In the present work, we developed and characterized a novel red FP-based optogenetic module, which undergoes proteolytic degradation upon illumination with non-phototoxic 567 nm light. The performance of the engineered OT in mammalian cells was validated via the activation of the luciferase reporter, Western blot analysis and its coupling to a transactivator via the nanobody–antigen interaction. This developed OT enabled spectral multiplexing with probes activatable by blue and far-red light. Our work expands the toolbox of OTs based on the GFP-like protein family, which do not require exogenous chromophores and introduces the first photodegradable red FP-based optogenetic module. 

## 2. Results

### 2.1. Mutagenesis and Screening of FusionRed Mutants

To develop a small optogenetic module with autocatalytically formed chromophore for spectral multiplexing, we selected FusionRed red FP as a template because of its minimal absorption at ~450 nm and above 650 nm, and the wavelengths were used for the regulation of blue and far-red/NIR light-sensing OTs [32]. We targeted via random mutagenesis a short amino acid region consisting of β-strands 7 and 8, which are close to the chromophore and comprise the A/C conservative dimerization interface of GFP-like proteins. A relatively low number of mutants due to the short length of the mutated portion allowed us to analyze them via screening in live mammalian cells.

**Figure 1:**
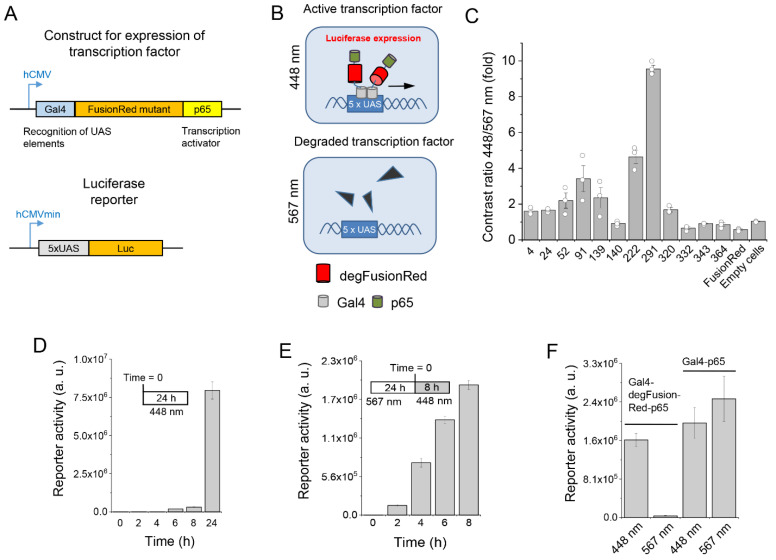
Screening of FusionRed mutants via light-controlled transcription. (**A**) Genetic constructs for the expression of FusionRed mutants in HEK293 cells. FusionRed gene is placed in the frame between Gal4 and p65 domains under the human CMV promoter (top). The *Gaussia* luciferase gene is placed under the control of the human CMV minimal promoter and 5×UAS operator region (bottom). (**B**) Schematics of Gal4-degFusionRed-p65 light regulation in mammalian cells. Under 448 nm light, two Gal4 domains of the Gal4-degFusionRed-p65 transcription factor recognize 5×UAS elements of the reporter construct and activate its transcription (top). The 567 nm light inactivates Gal4-degFusionRed-p65 (bottom). (**C**) Comparison of 12 selected clones by 448 nm/567 nm contrast of bioluminescent signal from the luciferase reporter; the mass ratio of the mutant-encoding and reporter plasmids is 4:1. (**D**) Increase in luciferase activity with time upon 448 nm illumination. (**E**) Increase in reporter activity upon 448 nm illumination after 24 h treatment with 567 nm light. (**F**) Comparison of performance of the Gal4- and p65-containing transcription factors with and without degFusionRed optogenetic module.

For this, we developed a transcription regulatory system expressing a *Gaussia* luciferase reporter naturally having a secretion motif (Figure 1A). Mutated FusionRed sequences were placed between transcription factor Gal4 and transactivator p65 and expressed under the control of the CMV promoter. The reporter construct activated by the resulting Gal4-FusionRedmutant-p65 fusions had the minimal CMVmin promoter controlled by 5×UAS repeats upstream of the luciferase. The expression of a FusionRed mutant coupled to Gal4 and p65 should have resulted in the activation of luciferase reporter expression and secretion in the cultured medium (Figure 1B).

HEK293 cells cotransfected with the individual FusionRed mutants and reporter were cultured in multiwell plates in duplicate and one plate was illuminated with 448 nm while another, with the copies of the mutant clones, was illuminated with 567 nm light, and a difference in luciferase activities for each clone was assessed. About 10^4^ mutant clones were screened.

We found that upon 567 nm illumination, some FusionRed mutants promoted decreased luciferase production as compared to 448 nm illumination. Figure 1C summarizes the performance of the clones in which 567 nm light caused the decrease in the luciferase expression. Clone 291 outperformed the others and exhibited ~10-fold photoinhibition of the reported production by yellow-orange light as compared to the blue light. The 291 clone was later (see below) named a degradable FusionRed (degFusionRed) and selected for further characterization.

### 2.2. Optimization of Transcription with degFusionRed

We found that the mass ratio between the Gal4-degFusionRed-p65 and luciferase plasmids with the total amount of DNA being constant in cell cotransfection affected the reporter production in both the 448 and 567 nm illuminated HEK293 cells (Supplementary Figure S1A). The plasmid ratio of 4:1, producing the highest luciferase activity difference between the 448 nm and 567 nm illuminated cells 48 h after the cotransfection, was used in further experiments.

When a fixed amount of reporter plasmid was used for transfection and the amount of the added Gal4-degFusionRed-p65-encoding plasmid gradually increased, the 567 nm light-induced reporter inhibition first grew and then decreased (Supplementary Figure S2A). However, shorter 567 nm light pulses (30 s) caused a higher degree of luciferase reporter inhibition and at lower Gal4-degFusionRed-p65 concentrations than the longer ones (120 s) (Supplementary Figure S2A,B). The bell-like pattern of the reporter inhibition persisted for various illumination regimens, suggesting that higher Gal4-degFusionRed-p65 expression might lead to self-inhibition by aggregation or the molecular crowding effect.

Under the light, luciferase activity becomes detectable in the cultured medium as soon as 6–8 h after cotransfection (Figure 1D, Supplementary Figure S1B). When HEK293 cells were first pre-incubated for 24 h under 567 nm light and then transferred to 448 nm light, the reporter was already detected 2 h under 448 nm light and then stably increased to ~14-fold during a further 8 h of experiment (Figure 1E). This suggests that after ending the exposure to 567 nm light, the production of its new molecules occurred de novo via protein synthesis. Likely, 567 nm light caused a substantial reduction in the Gal4-degFusionRed-p65 transcription factor in cells. Indeed, when the luciferase reporter was cotransfected with a Gal4-p65-encoding plasmid lacking the degFusionRed insert, the luciferase expression was not inhibited by 567 nm light (Figure 1F), which excluded the possibility of a non-specific effect of light on protein synthesis.

### 2.3. Biochemical and Spectral Characterization of degFusionRed

**Figure 2:**
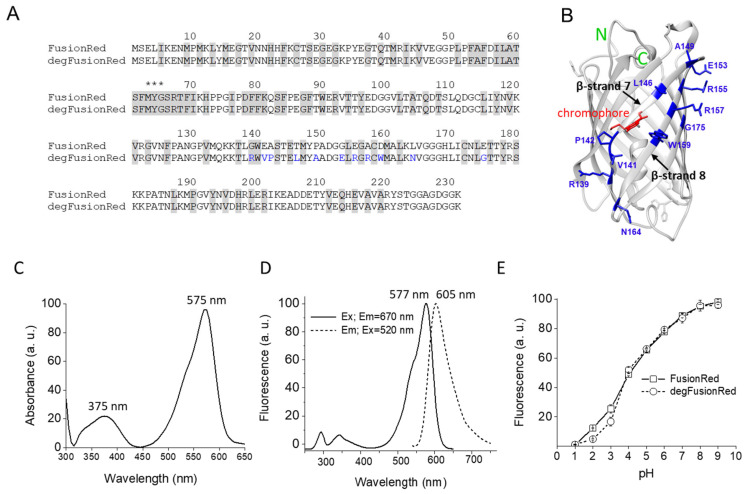
Mutations of degFusionRed and its spectral and biochemical characterization. (**A**) Alignment of FusionRed and degFusionRed amino acid sequences. Amino acid residues whose side chains oriented inside of the protein β-can are highlighted gray. Mutations in the degFusionRed sequence relative to parental FusionRed are blue. Amino acid residues, which autocatalytically form a chromophore, are marked with asterisks. (**B**) Crystal structure of FusionRed (PDB ID: 6U1A) in which 11 amino acid residues were computationally swapped with corresponding amino acid substitutions of degFusionRed (marked blue). Residue swap was performed using UCSF Chimera 1.16 software. (**C**) Absorbance spectrum of purified degFusionRed. (**D**) Fluorescence excitation (determined at 670 nm emission) and emission spectra (determined at 520 nm excitation) of purified degFusionRed. (**E**) pH dependencies of purified degFusionRed and parental FusionRed.

Compared to parental FusionRed protein, degFusionRed contained 11 amino acid residue substitutions: G139R, E141V, A142P, T146L, P149A, G153E, E155R, A157R, D159W, L164N and E175G (Figure 2A), of which 10 were located in β-strands 7 and 8, and the last one, unexpectedly, occurred in β-strand 9 (Figure 2B). The absorption spectrum of degFusionRed had maxima at 375 nm and 575 nm and had a minimum at ~450 nm (Figure 2C). It exhibited the fluorescence excitation and emission maxima at 577 nm and 605 nm, respectively (Figure 2D), which were slightly blue-shifted as compared to parental FusionRed (580 nm and 608 nm, respectively) [32]. The pH stability of degFusionRed was similar to that of FusionRed [32], with the p*K_a_* of ~4.5 (Figure 2E).

### 2.4. Mode of Action of degFusionRed

**Figure 3:**
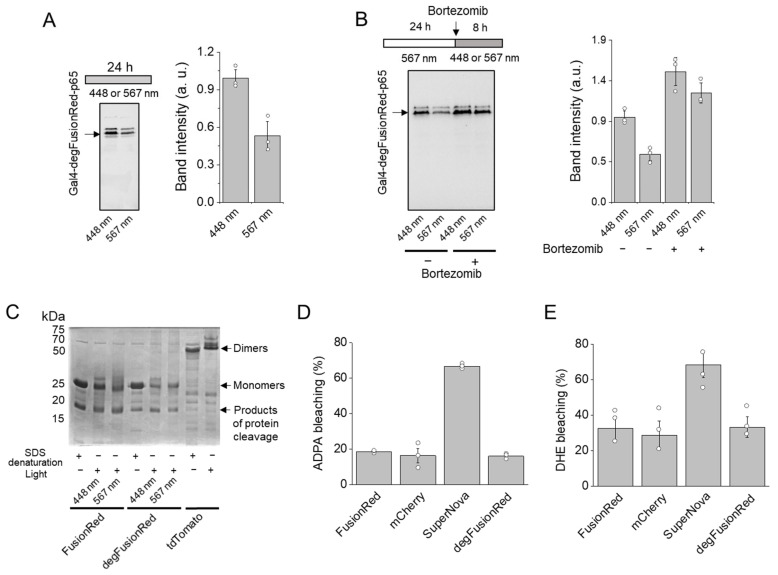
Analysis of the mode of action of degFusionRed. (**A**) Effect of 567 nm light on the amount of Gal4-degFusionRed-p65 transcription factor studied via Western blot in HEK293 cell lysate. Cells were illuminated with 448 nm or 567 nm light for 24 h after transfection. The right graph shows the quantification of intensities of the Western blot Gal4-degFusionRed-p65 bands. (**B**) Effect of bortezomib on Gal4-degFusionRed-p65 transcription factor in HEK293 cells evaluated via Western blot. After 567 nm cell illumination for 24 h, cells with or without the addition of bortezomib were treated with 448 nm light or kept under 567 nm for an additional 8 h. The right graph shows the quantification of intensities of the Western blot Gal4-degFusionRed-p65 bands. (**C**) Semi-native PAGE gel analysis of FusionRed, degFusionRed and tdTomato dimeric control purified from bacteria after 8% denaturation with 8% SDS, 488 nm or 567 nm illumination. (**D**) Singlet oxygen production in purified FusionRed, mCherry, SuperNova and degFusionRed protein samples determined via photobleaching of ADPA. (**E**) Superoxide production in purified FusionRed, mCherry, SuperNova and degFusionRed protein samples determined via photobleaching of DHE.

We next studied HEK293 cell lysates using Western blot with polyclonal anti-tRFP antibodies that recognize both native and denatured forms of all TagRFP-derived FPs, including FusionRed. We found that 567 nm illumination for 24 h resulted in a notable decrease in degFusionRed in HEK293 cells compared to 448 nm illumination (Figure 3A). When cells were pre-illuminated with 567 nm light for 24 h and then remained under this light for an additional 8 h or were transferred to 448 nm light with the addition of a proteasomal inhibitor bortezomib, Western blot demonstrated the abolishment of the 567 nm light-induced reduction in degFusionRed amount (Figure 3B), indicating that 567 nm illumination induced the proteasomal degradation of our optogenetic module. However, a sufficient amount of degFusionRed was still detected after 8 h, suggesting that before being completely degraded, degFusionRed remains in the inactive unfolded state, which should still be recognized according to the antibody manufacturer (see Section 4).

To determine whether light caused the oligomerization of degFusionRed, we further applied it to a semi-native PAGE (without heating the samples). Parental FusionRed, degFusionRed and tdTomato [33] were expressed and purified from TOP10 bacteria. The non-illuminated protein denaturation control contained the proteins denatured with 8% SDS. The tdTomato dimer was used as a dimeric control. We found that both degFusionRed and parental FusionRed proteins exhibited monomeric behavior upon illumination with either 448 nm or 567 nm light (Figure 3C), excluding the possibility of light-induced degFusionRed dimerization or oligomerization.

We further studied whether reactive oxygen species (ROS) are generated by degFusionRed. For this, we compared degFusionRed with known ROS generator SuperNova FP [29] and commonly used red FPs, such as parental FusionRed [32] and mCherry [34]. All FPs were expressed and purified from TOP10 bacteria and applied to 530 nm light. The illumination of SuperNova induced the photobleaching of anthracene-9,10-dipropionic acid (ADPA), an indicator of singlet oxygen, ~4-fold more efficiently than degFusionRed. At the same time, no differences between ADPA photobleaching caused by degFusionRed, FusionRed and mCherry were observed (Figure 3D). The photobleaching of dihydroethidium (DHE), an indicator of superoxide ion, by SuperNova was ~2.5-fold more efficient than by degFusionRed (Figure 3E). Similarly, there was no difference between DHE photobleaching by degFusionRed, FusionRed or mCherry. This indicated that ROS production by degFusionRed unlikely induced CALI of fused domains, in contrast to what was observed for KillerRed [30], SuperNova [29] and miniSOG [31] ROS molecular generators.

Overall, these data suggest that the mechanism of the degFusionRed action is proteasomal degradation, likely upon its light-induced conformational changes resulting in ubiquitination.

### 2.5. Analysis of degFusionRed Spectral Compatibility

**Figure 4:**
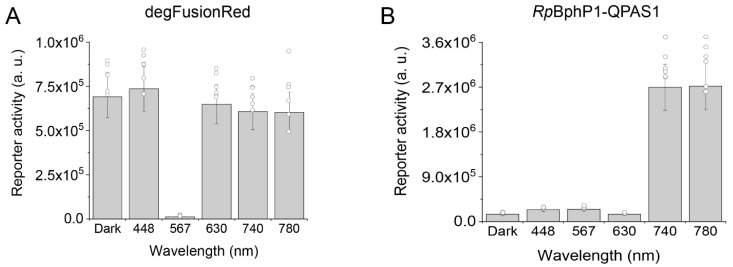
Spectral sensitivity of degFusionRed and *Rp*BphP1-QPAS1 optogenetic systems. (**A**) Performance of Gal4-degFusionRed-p65 transcription factor in HEK293 cell under 448 nm, 567 nm, 630 nm, 740 nm and 780 nm light determined by the activity of cotransfected luciferase reporter construct depicted in Figure 1A (bottom). (**B**) Performance of *Rp*BphP1-VP16 and Gal4-QPAS1 heterodimerizing optogenetic pair in HEK293 cell under 448 nm, 567 nm, 630 nm, 740 nm and 780 nm light determined by the activity of cotransfected luciferase reporter construct depicted in Figure 1A (bottom).

We further studied the possibility of the future spectral multiplexing of degFusionRed. In HEK293 cells, we found that 448 nm, 630 nm, 740 nm and 780 nm light did not cause the inhibition of luciferase production via our optogenetic module (Figure 4A) as determined via bioluminescence assay. The *Gaussia* luciferase reporter activity at these wavelengths was not statistically different from that in the darkness. In parallel, we coexpressed in HEK293 a known *Rp*BphP1-QPAS1 optogenetic system, which is controlled with far-red/NIR light, and the same luciferase reporter plasmid. As expected, 740 nm and 780 nm activated the luciferase production by the *Rp*BphP1-QPAS1 system, whereas the darkness, 448 nm, 567 nm and 630 nm did not cause substantial bioluminescence signal (Figure 4B). This indicated the spectral compatibility of degFusionRed (567 nm) with *Rp*BphP1-QPAS1 (740–780 nm), as well as with blue (448 nm) light-activatable OTs, thus potentially enabling the spectral multiplexing of three distinct OTs in mammalian cells.

### 2.6. Nanobody-Coupled degFusionRed for Protein Control

**Figure 5:**
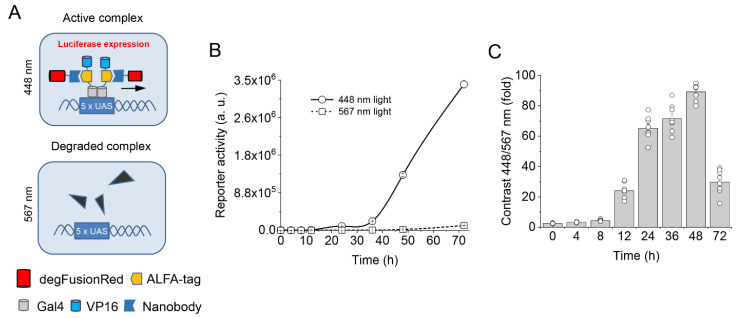
Light-dependent degradation of protein of interest by degFusionRed. (**A**) A model protein of interest (Gal4-ALFA-tag-VP16) targeted by anti-ALFA-tag nanobody fusion with degFusionRed (Nb_ALFA_-degFusionRed) to form a complex. Under 448 nm light (top), the formed transcription complex binds 5×UAS, resulting in the transcription of the luciferase reporter. Under 567 nm light (bottom), the Gal4-ALFA-tag-VP16 transcription factor is degraded together with the bound Nb_ALFA_-degFusionRed. (**B**) Kinetics of luciferase expression in HEK293 cells under 448 nm and 567 nm illumination from the beginning of transfection to 72 h of the experiment. (**C**) The difference in the luciferase expression between 448 nm and 567 nm illuminated cells measured in panel (**B**) results in large contrast values, reaching its maximum at 48 h after cell transfection.

We next studied whether we could photodegrade a POI by targeting it with degFusionRed fused to a nanobody specific for this protein. Nanobodies are single variable domains on heavy chain (VHH) antibodies of camelids. Nanobodies are small (15 kDa) and fully genetically encoded, and can recognize cognate antigens with high affinity and specificity. As a model POI, we used an engineered transcription factor consisting of Gal4-ALFA-tag-VP16 fusion. In this POI, the ALFA-tag peptide [35] was placed between the Gal4 DNA-binding domain and VP16 transactivator. In turn, degFusionRed was fused with an anti-ALFA-tag nanobody (Nb_ALFA_), so that upon co-expression of both fusions, degFusionRed would be coupled with the POI via non-covalent antigen–nanobody interaction (Figure 5A). To detect light-induced changes in the POI level, we cotransfected the third plasmid, encoding the *Gaussia* luciferase reporter after 5×UAS repeats (Figure 1A, bottom).

We found that under 448 nm light, the luciferase production was high, whereas 567 nm light caused a substantial decrease in the luciferase activity (Figure 5B, Supplementary Figure S3), indicating a reduction in the Gal4-ALFA-tag-VP16 level caused by the yellow-orange illumination.

To characterize this OT further, we varied the transfection conditions by optimizing the mass ratio of plasmids encoding Gal4-ALFA-tag-VP16, Nb_ALFA_-degFusionRed, and luciferase reporter (Supplementary Figure S3). We found that the mass ratio of 10:80:10 of these cotransfected plasmids resulted in the highest decrease in the luciferase reporter production, which indicated the significant downregulation of the POI (Gal4-ALFA-tag-VP16) targeted by Nb_ALFA_-degFusionRed.

We next studied the time course of the luciferase reporter activity under pulsed 567 nm and 488 nm (control) illumination. The luciferase activity became apparent ~12 h after co-transfection under 448 nm light and accumulated almost linearly after 36 h (Figure 5B), indicating the continuous presence of the Gal4-ALFA-tag-VP16 protein. In contrast, the 567 nm illumination caused a significant decrease in the luciferase activity readout during all studied periods (until 72 h), which is in line with the continuous degradation of this model POI in cells.

Because our OT can also be used as the light-regulated transcription inhibition system, it was interesting to determine the efficiency of its performance. Indeed, the maximum difference in luciferase activity readouts between the 448 nm and 567 nm illuminated cells was ~90–95-fold and was achieved 48 h after cotransfection (Figure 5C). Notably, this difference is significantly larger than that reported for the far-red/NIR *Rp*BphP1-QPAS1 pair (42-fold activation [2]), which is also a two-component OT. The observed overperformance of the degFusionRed-based OT can be attributed to its several-fold smaller size, resulting in its higher expression level, and to its efficiently autocatalytically formed chromophore, unlike an exogenous biliverdin chromophore of *Rp*BphP1 to which *Rp*BphP1 has a low binding affinity.

## 3. Discussion

We engineered, for the first time, the photodegradable variant of FusionRed red fluorescent protein, degFusionRed, via random mutagenesis of its 7 and 8 β-strands, which are involved in the conserved A/C dimerization interface of GFP-like proteins. The degFusionRed preserved the major characteristics of parental FusionRed including spectral characteristics, which should allow for its simultaneous use with blue and far-red/NIR light-sensing OTs for the regulation of complex molecular and physiological processes at multiple control points.

Unlike the parental protein, degFusionRed is degraded by yellow-orange light in mammalian cells, likely via the proteasomal pathway, as evidenced via Western blot analysis of samples treated with proteasomal inhibitor bortezomib. In contrast to KillerRed [30], SuperNova [29] and miniSOG [31] ROS photosensitizers, degFusionRed preserves the characteristics of parental FusionRed in terms of low ROS production.

Mutations found in degFusionRed may result in light-induced structural rearrangements of the 7 and 8 β-strands. Due to the high rigidity of proline residues [36], A142P substitution may affect the H-bond existing between the chromophore’s phenolate group and neighboring Ser143 [37]. Additionally, a water-mediated H-bond between the chromophore’s phenolate of FusionRed and the main chain carbonyl of Glu141 can be affected by both neighboring mutations A142P and E141V. The substitution of P149A in β-strand 7 may confer increased flexibility or destabilization of the β-strand. The hydrophobic R-group of Trp introduced instead of negatively charged Asp159 tends to burrow into the hydrophobic protein core, which can also destabilize β-strand 8.

Structural rearrangements induced by 567 nm light in degFusionRed may be similar to that of photodissociable FP Dronpa. In the latter, light induces chromophore cis-*trans* isomerization and the disruption of intramolecular bonds between the chromophore’s phenolate and β-can, leading to the increased flexibility of the neighboring β-strands 7–10 and subsequent dimer disruption [26,27]. Ser143 and Glu141 of FusionRed correspond to Ser142 and Glu140 of Dronpa and also form H-bonds with the chromophore’s phenolate. In degFusionRed, 567 nm light may enhance the flexibility of the chromophore-adjacent β-can portion, leading to an exposition of the inner hydrophobic patches, which can be recognized by ubiquitin ligases [28].

The nanobody-coupled degFusionRed can effectively degrade the transcription factor tagged with a nanobody-specific antigenic peptide, indicating that other intracellular POIs can be specifically eliminated by yellow-orange light [38].

Current approaches for the regulation of POI degradation have both advantages and limitations. The application of small exogenous molecules, such as molecular glues [39], proteolysis targeting chimeras (PROTACs) [40,41] and auxin-inducible degrons (AIDs) [42,43] is an effective chemogenetic technique; however, they have lower spatial and temporal accuracy compared to OTs. Additionally, compared to protein degradation via proteases and OTs, small molecules are not genetically encoded, which does not allow for performing non-invasive experiments. Similarly, the Trim-Away approach for targeted POI degradation [44] is used for the effective elimination of POI but requires the microinjection of these antibodies directly into cells.

The application of proteases alone may lack effective POI regulation. For instance, TEV protease-induced protein inactivation (TIPI) [45] can be used to study cell processes if both the TEV and TIPI degron are combined in a cell or intracellular compartment. Similarly, bacterial degron moieties targeted by ATP-dependent prokaryotic proteases (Clp family, Lon and AAA+) [46] can be used in bacterial cells or mitochondria but not in the cytosol of mammalian cells.

Optogenetic approaches, such as the LOV2-caging of degrons [47,48], cleaving degrons off via the LOV2-caged TEV protease site [49] and degradable CRY2 oligomers [50], provide non-invasive and efficient temporal and spatial POI regulation; however, they are regulated with blue light, which does not allow for multiplexing with other LOV-based OTs.

Compared to the other approaches, degFusionRed enables the optogenetic regulation of POIs via a previously unexplored yellow-orange spectral range, independence from cofactors, such as biliverdin or PCB, and spectral multiplexing with blue and NIR light-controlled OTs.

In conclusion, we developed a small versatile photodegradable optogenetic module degFusionRed with autocatalytic chromophore formation and demonstrated the effective photoinhibition of gene transcription and targeted protein photodegradation in mammalian cells, thus spectrally expanding the toolbox of genetically encoded OTs.

## 4. Materials and Methods

### 4.1. Random Mutagenesis

A construct for mammalian expression of Gal4-FusionRed-p65 fusion was created via the insertion of residues 2–148 of the yeast transcription activator Gal4 into the pEGFP-N1 backbone via NheI/BglII sites and subsequent substitution of EGFP-encoding sequence by AgeI/Not sites with the encoding residues 428–551 of human RELA protein (p65 domain). The resulting construct was amplified via polymerase chain reaction (PCR) using high-fidelity DNA polymerase PrimeSTAR Max (Takara Bio, Shiga, Japan), excluding the nucleotide segment corresponding to the region Val132–Asn173 of FusionRed FP, which comprised the 7 and 8 β-strands. The latter 126 bp DNA portion was amplified via error-prone PCR using a GeneMorph II random mutagenesis kit (Agilent Technologies, Santa Clara, CA, USA) and inserted via blunt-end ligation in the amplified vector, containing the rest parts of the coding sequence. Ligated DNA mixture was purified using QIAprep 2.0 Spin Columns (Qiagen, Hilden, Germany) and used for electroporation of TOP10 *E. coli* cells (Invitrogen, Waltham, MA, USA). Bacteria were plated on LB agar with 100 µg/mL kanamycin. Constructs with the correct insert orientation were selected via PCR analysis.

### 4.2. Mammalian Cell Culture and Transfection

HEK293 cells were obtained from ATCC (CRL-1573). Cells were cultured in Dulbecco’s Modified Eagle Medium (DMEM) with 4 g/L glucose, 4 mM L-glutamine, 10% FBS (Life Technologies, Invitrogen, Waltham, MA, USA) and penicillin-streptomycin mixture (Life Technologies, Invitrogen, Carlsbad, CA, USA) at 37 °C in a humidified CO_2_ incubator with 5% CO_2_. Transfection was performed via the application of polyethyleneimine (PEI) [51]. For mock transfection, the *Gaussia* luciferase reporter pGL3-Basic plasmid (Promega, Madison, WI, USA) with the luciferase gene removed was used.

### 4.3. Screening of Mutants in Mammalian Cells

The day before transfection, cells were trypsinized and transferred into wells of polystyrene 96-well culturing plates (Costar). Ten µL of a water solution containing 30 ng of the mutated Gal4-FusionRed-p65 construct, 0.1 µg of the pG5-Luc reporter (Promega), encoding the secreted *Gaussia* luciferase, and 0.2 µg pGl3-Basic vector was mixed with 1.5 µL of 1 mg/mL solution of PEI, pH = 7.0. The mixture was incubated for 10 min at room temperature for the efficient formation of DNA-PEI complexes and added directly to cells in plate wells containing 100 µL of the cultured medium. After transfection, cells were illuminated for 24 h with either blue pulsed light (array of 448 nm light-emitting diodes (LEDs), 30 s ON, 5 min OFF) or yellow-orange pulsed light (567 nm LED array, 30 s ON, 5 min OFF) at a light power density of 100 mW/cm^2^.

For measurements of *Gaussia* luciferase activity, 1 µL of cell culture medium was mixed with 100 µL of 2 µM coelenterazine (Sigma, St. Louis, MO, USA) in wells of 96-well reflective bottom plates for bioluminescent assays (Stellar Scientific, Owings Mills, MD, USA). The quantification of bioluminescence was performed using a Victor X-5 plate reader (Perkin Elmer, Waltham, MA, USA). The contrast of 448 nm/567 nm reporter expression was calculated as luciferase activity in samples under illumination with 448 nm light divided by that at 567 nm light.

### 4.4. Protein Expression and Purification

Nucleotide sequences of FusionRed, degFusionRed, mCherry and tdTomato proteins were inserted via BglII/EcoRI sites to a pBAD/His-B vector (Invitrogen), which was previously modified [52] to minimize a linker between N-terminal hexahistidine tag and the coding sequence (also known as pBAD/His-D). TOP10 *E.coli* cells transformed with corresponding constructs were cultivated to OD_600_ = 0.5, and protein expression was induced by the addition of 0.002% arabinose. After incubation for 3 h at 37 °C upon vigorous shaking, bacteria were pelleted via centrifugation at 5000× *g* for 15 min at 4 °C (Eppendorf 5810 R Centrifuge) and used directly for the protein purification protocol or frozen at −20 °C.

The SuperNova gene in the pRSET_B_ vector [29] was transformed in BL21(DE3) *E. coli* strain (Invitrogen). Bacteria were cultured at 37 °C to OD_600_ = 0.5, and induction was performed by the addition of 0.05 mM IPTG. After incubation for 3 h at 30 °C upon vigorous shaking, bacteria were pelleted and used for protein purification.

After being resuspended in ice-cold phosphate-buffered saline (PBS), bacterial cells were disrupted using an ultrasound homogenizer (Model 3000 Ultrasonic Homogenizer; BioLogics). The soluble fraction of cell lysates was applied to Ni-NTA agarose (Macherey-Nagel, Dueren, Germany) columns equilibrated with a 10 mM solution of imidazole in PBS. Proteins were eluted by 400 mM imidazole in PBS and dialyzed overnight in 1000× volume of PBS.

### 4.5. Absorbance and Fluorescence Spectroscopy

Protein samples were diluted in PBS to the concentration of 0.1–0.5 mg/mL. Absorption spectra were recorded using the Hitachi U-2000 spectrophotometer. Fluorescence spectra were recorded using the Cary Varian Eclipse Fluorescence Spectrophotometer (Agilent Technologies, Santa Clara, CA, USA).

### 4.6. Western Blot Analysis

The day before transfection, cells were trypsinized and transferred into 9.5 cm^2^ cell culture dishes (Costar, Washington, DC, USA). The transfection mix contained 30 µg of the plasmid encoding Gal4-degFusionRed-p65 and 150 µg PEI in 1 mL PBS, which was added to 10 mL of culturing medium in dishes. After transfection, cells were illuminated for 24 h with blue pulsed light (448 nm LED array, 30 s ON, 5 min OFF) and by pulsed light (567 nm LED array, 30 s ON, 5 min OFF) at a light power density of 100 mW/cm^2^.

Then, cells were detached using a cell scraper (Falcon) and disrupted by passing them 10 times through the 27G needle in 0.5 mL of 10 mM HEPES, 10 mM KCl, 1 mM MgCl_2_ and 1 mM EDTA, pH = 7.2, with the addition of complete mini protease inhibitors (Roche Diagnostics, Mannheim, Germany). Cell homogenate was centrifuged at 700 *g* for 5 min to pellet the nuclei. The cytoplasmic extract was centrifuged again at 15,000 *g* for 5 min to remove organelles, and then combined with 4x Laemli Sample buffer and boiled for 3 min. Proteins from the resulting samples were separated via 12% SDS-PAGE, transferred to a nitrocellulose membrane and analyzed using polyclonal anti-tRFP antibodies which recognize both the native and denatured form of FusionRed (Evrogen, #AB233, Moscow, Russia) in a dilution of 1:2000, and polyclonal goat anti-rabbit IgG HRP conjugate (Thermo Fisher, #32460, Waltham, MA, USA) in a dilution of 1:2000. The enhanced chemiluminescent (ECL) approach [53] was used for the detection of protein bands.

### 4.7. Semi-Native PAGE Gel

Proteins were diluted to the concentration of 2 mg/mL in PBS and illuminated either with constant blue light (448 nm LED array) or yellow-orange light (567 nm LED array) for 0.5 h. Twelve µg of protein samples was diluted in 4× loading buffer (250 mM Tris-HCl pH 6.8, 0.008% bromophenol blue, 4% glycerol) or in the same loading buffer but with the addition of 8% SDS for denaturation control. The samples were then loaded on 4–20% gradient gel (BioRad, Hercules, CA, USA). The protein samples were not heated but the running buffer contained 1% SDS. After 2 h of electrophoresis, the gel was washed and stained with GelCode blue protein stain (BioRad, Hercules, CA, USA).

### 4.8. Photobleaching Assay

For the evaluation of singlet oxygen production via FPs, samples containing 0.5 mg/mL of respective proteins and 10 μM ADPA in PBS were illuminated for 10 min with constant 1 W/cm^2^ of 530 nm light of LED array. The production of superoxide was evaluated via the illumination of samples containing 0.5 mg/mL of proteins and 0.1 mM DHE in PBS for 2 min with constant 1 W/cm^2^ of 530 nm light. The registration of the fluorescence of ADPA and DHE change in fluorescence intensity (photobleaching) was performed using the Victor X5 plate reader with 350 nm fluorescence excitation and 450 nm emission filters.

### 4.9. Spectral Multiplexing with RpBphP1-QPAS1

For the characterization of spectral multiplexing suitability, HEK293 cells in 24-well plates were cotransfected with pG5-Luc reporter plasmid, and a plasmid encoding Gal4-degFusionRed-p65, or with pQP-T2A [2] plasmid-expressing *Rp*BphP1-VP16 fusion and Gal4-QPAS1 fusion in a mass ratio of 1:3. Then, cells were cultivated for 24 h in the darkness or under the pulsed light (30 s ON, 3 min OFF) of different wavelengths. The light was produced by arrays of 448, 567, 630, 740 and 780 nm LEDs. After illumination, the activity of secreted luciferase in the cell supernatant was measured.

### 4.10. Light Regulation of Gal4-ALFA-Tag-VP16 via Nanobody-degFusionRed

HEK293 cells in 96-well plates were cotransfected with plasmids encoding Gal4-ALFA-tag-VP16, nanobody to ALFA-tag [35,54] (Nb_ALFA_) fused to degFusionRed and *Gaussia* reporter plasmid pG5-Luc. Titration from 1:89 to 80:1 was performed to determine the optimal mass ratio of plasmids encoding Gal4-ALFA-tag-VP16 and Nb_ALFA_-degFusionRed plasmids. Either 1 or 10 parts of the pG5-Luc reporter were used for titration. Cells were cultured for 24 h after cotransfection under either 448 nm or 567 nm light, and then luciferase activity was assessed. For the time course of luciferase production, the mass ratio of 8:1:1 for these plasmids was used. For this, the cotransfected cells were cultured for 72 h under either 448 nm or 567 nm light and luciferase activity was measured at 0, 4, 8, 12, 24, 36, 48 and 72 h after the cotransfection.

## Data Availability

The main data supporting the findings of this study are available within the article and its Supplementary Materials. Additional data are available from the corresponding author upon request.

## Supplementary Materials

The supporting information can be downloaded at https://www.mdpi.com/article/10.3390/ijms24076526/s1.

## References

[1] Müller K., Engesser R., Schulz S., Steinberg T., Tomakidi P., Weber C.C., Ulm R., Timmer J., Zurbriggen M.D., Weber W. (2013). Multi-chromatic control of mammalian gene expression and signaling. Nucleic Acids Res..

[2] Redchuk T.A., Omelina E.S., Chernov K.G., Verkhusha V.V. (2017). Near-infrared optogenetic pair for protein regulation and spectral multiplexing. Nat. Chem. Biol..

[3] Redchuk T.A., Karasev M.M., Verkhusha P.V., Donnelly S.K., Hülsemann M., Virtanen J., Moore H.M., Vartiainen M.K., Hodgson L., Verkhusha V.V. (2020). Optogenetic regulation of endogenous proteins. Nat. Commun..

[4] Chernov K.G., Redchuk T.A., Omelina E.S., Verkhusha V.V. (2017). Near-Infrared Fluorescent Proteins, Biosensors, and Optogenetic Tools Engineered from Phytochromes. Chem. Rev..

[5] Zhang K., Cui B. (2015). Optogenetic control of intracellular signaling pathways. Trends Biotechnol..

[6] Zhang W., Lohman A.W., Zhuravlova Y., Lu X., Wiens M.D., Hoi H., Yaganoglu S., Mohr M.A., Kitova E.N., Klassen J.S. (2017). Optogenetic control with a photocleavable protein, PhoCl. Nat. Methods.

[7] Losi A., Gardner K.H., Möglich A. (2018). Blue-Light Receptors for Optogenetics. Chem. Rev..

[8] Polstein L.R., Gersbach C.A. (2012). Light-inducible spatiotemporal control of gene activation by customizable zinc finger transcription factors. J. Am. Chem. Soc..

[9] Salinas F., Rojas V., Delgado V., López J., Agosin E., Larrondo L.F. (2018). Fungal Light-Oxygen-Voltage Domains for Optogenetic Control of Gene Expression and Flocculation in Yeast. mBio.

[10] Benedetti L., Marvin J.S., Falahati H., Guillén-Samander A., Looger L.L., De Camilli P. (2020). Optimized Vivid-derived Magnets photodimerizers for subcellular optogenetics in mammalian cells. eLife.

[11] Strickland D., Lin Y., Wagner E., Hope C.M., Zayner J., Antoniou C., Sosnick T.R., Weiss E.L., Glotzer M. (2012). TULIPs: Tunable, light-controlled interacting protein tags for cell biology. Nat. Methods.

[12] Bugaj L.J., Choksi A.T., Mesuda C.K., Kane R.S., Schaffer D.V. (2013). Optogenetic protein clustering and signaling activation in mammalian cells. Nat. Methods.

[13] Chen Z., Kang X.W., Zhou Y., Zhou Z., Tang S., Zou S., Wang K., Huang J., Ding B., Zhong D. (2023). Dissecting the Ultrafast Stepwise Bidirectional Proton Relay in a Blue-Light Photoreceptor. J. Am. Chem. Soc..

[14] Ando R., Mizuno H., Miyawaki A. (2004). Regulated fast nucleocytoplasmic shuttling observed by reversible protein highlighting. Science.

[15] Zhou X.X., Fan L.L.Z., Li P.P., Shen K., Lin M.Z. (2017). Optical control of cell signaling by single-chain photoswitchable kinases. Science.

[16] Schneider N., Chatelle C.V., Ochoa-Fernandez R., Zurbriggen M.D., Weber W. (2021). Green Light-Controlled Gene Switch for Mammalian and Plant Cells. Methods Mol. Biol..

[17] Jang J., McDonald S., Uppalapati M., Woolley G.A. (2019). Green, orange, red, and far-red optogenetic tools derived from cyanobacteriochromes. bioRxiv.

[18] Song P., Yang Z., Guo C., Han R., Wang H., Dong J., Kang D., Guo Y., Yang S., Li J. (2023). 14-3-3 proteins regulate photomorphogenesis by facilitating light-induced degradation of PIF3. New Phytol..

[19] Ochoa-Fernandez R., Abel N.B., Wieland F.G., Schlegel J., Koch L.A., Miller J.B., Engesser R., Giuriani G., Brandl S.M., Timmer J. (2020). Optogenetic control of gene expression in plants in the presence of ambient white light. Nat. Methods.

[20] Yu Y., Wu X., Guan N., Shao J., Li H., Chen Y., Ping Y., Li D., Ye H. (2020). Engineering a far-red light-activated split-Cas9 system for remote-controlled genome editing of internal organs and tumors. Sci. Adv..

[21] Kuwasaki Y., Suzuki K., Yu G., Yamamoto S., Otabe T., Kakihara Y., Nishiwaki M., Miyake K., Fushimi K., Bekdash R. (2022). A red light–responsive photoswitch for deep tissue optogenetics. Nat. Biotechnol..

[22] Kaberniuk A.A., Baloban M., Monakhov M.V., Shcherbakova D.M., Verkhusha V.V. (2021). Single-component near-infrared optogenetic systems for gene transcription regulation. Nat. Commun..

[23] Kaberniuk A.A., Shemetov A.A., Verkhusha V.V. (2016). A bacterial phytochrome-based optogenetic system controllable with near-infrared light. Nat. Methods.

[24] Kciuk M., Marciniak B., Mojzych M., Kontek R. (2020). Focus on UV-Induced DNA Damage and Repair-Disease Relevance and Protective Strategies. Int. J. Mol. Sci..

[25] Bauer R., Hoenes K., Meurle T., Hessling M., Spellerberg B. (2021). The effects of violet and blue light irradiation on ESKAPE pathogens and human cells in presence of cell culture media. Sci. Rep..

[26] Andresen M., Stiel A.C., Trowitzsch S., Weber G., Eggeling C., Wahl M.C., Hell S.W., Jakobs S. (2007). Structural basis for reversible photoswitching in Dronpa. Proc. Natl. Acad. Sci. USA.

[27] Mizuno H., Mal T.K., Walchli M., Kikuchi A., Fukano T., Ando R., Jeyakanthan J., Taka J., Shiro Y., Ikura M. (2008). Light-dependent regulation of structural flexibility in a photochromic fluorescent protein. Proc. Natl. Acad. Sci. USA.

[28] Hermann A., Liewald J.F., Gottschalk A. (2015). A photosensitive degron enables acute light-induced protein degradation in the nervous system. Curr. Biol..

[29] Takemoto K., Matsuda T., Sakai N., Fu D., Noda M., Uchiyama S., Kotera I., Arai Y., Horiuchi M., Fukui K. (2013). SuperNova, a monomeric photosensitizing fluorescent protein for chromophore-assisted light inactivation. Sci. Rep..

[30] Bulina M.E., Chudakov D.M., Britanova O.V., Yanushevich Y.G., Staroverov D.B., Chepurnykh T.V., Merzlyak E.M., Shkrob M.A., Lukyanov S., Lukyanov K.A. (2006). A genetically encoded photosensitizer. Nat. Biotechnol..

[31] Torra J., Lafaye C., Signor L., Aumonier S., Flors C., Shu X., Nonell S., Gotthard G., Royant A. (2019). Tailing miniSOG: Structural bases of the complex photophysics of a flavin-binding singlet oxygen photosensitizing protein. Sci. Rep..

[32] Shemiakina I.I., Ermakova G.V., Cranfill P.J., Baird M.A., Evans R.A., Souslova E.A., Staroverov D.B., Gorokhovatsky A.Y., Putintseva E.V., Gorodnicheva T.V. (2012). A monomeric red fluorescent protein with low cytotoxicity. Nat. Commun..

[33] Shaner N.C., Campbell R.E., Steinbach P.A., Giepmans B.N., Palmer A.E., Tsien R.Y. (2004). Improved monomeric red, orange and yellow fluorescent proteins derived from *Discosoma* sp. red fluorescent protein. Nat. Biotechnol..

[34] Onukwufor J.O., Trewin A.J., Baran T.M., Almast A., Foster T.H., Wojtovich A.P. (2020). Quantification of reactive oxygen species production by the red fluorescent proteins KillerRed, SuperNova and mCherry. Free Radic. Biol. Med..

[35] Götzke H., Kilisch M., Martínez-Carranza M., Sograte-Idrissi S., Rajavel A., Schlichthaerle T., Engels N., Jungmann R., Stenmark P., Opazo F. (2019). The ALFA-tag is a highly versatile tool for nanobody-based bioscience applications. Nat. Commun..

[36] Yu H., Zhao Y., Guo C., Gan Y., Huang H. (2015). The role of proline substitutions within flexible regions on thermostability of luciferase. Biochim. Biophys. Acta.

[37] Muslinkina L., Pletnev V.Z., Pletneva N.V., Ruchkin D.A., Kolesov D.V., Bogdanov A.M., Kost L.A., Rakitina T.V., Agapova Y.K., Shemyakina I.I. (2020). Two independent routes of post-translational chemistry in fluorescent protein FusionRed. Int. J. Biol. Macromol..

[38] Marschall A.L., Dübel S. (2016). Antibodies inside of a cell can change its outside: Can intrabodies provide a new therapeutic paradigm?. Comput. Struct. Biotechnol. J..

[39] Dong G., Ding Y., He S., Sheng C. (2021). Molecular Glues for Targeted Protein Degradation: From Serendipity to Rational Discovery. J. Med. Chem..

[40] Carmony K.C., Kim K.B. (2012). PROTAC-induced proteolytic targeting. Methods Mol. Biol..

[41] Liu J., Chen H., Ma L., He Z., Wang D., Liu Y., Lin Q., Zhang T., Gray N., Kaniskan H. (2020). Light-induced control of protein destruction by opto-PROTAC. Sci. Adv..

[42] Yesbolatova A., Saito Y., Kitamoto N., Makino-Itou H., Ajima R., Nakano R., Nakaoka H., Fukui K., Gamo K., Tominari Y. (2020). The auxin-inducible degron 2 technology provides sharp degradation control in yeast, mammalian cells, and mice. Nat. Commun..

[43] Macdonald L., Taylor G.C., Brisbane J.M., Christodoulou E., Scott L., von Kriegsheim A., Rossant J., Gu B., Wood A.J. (2022). Rapid and specific degradation of endogenous proteins in mouse models using auxin-inducible degrons. eLife.

[44] Zeng J., Santos A.F., Mukadam A.S., Osswald M., Jacques D.A., Dickson C.F., McLaughlin S.H., Johnson C.M., Kiss L., Luptak J. (2021). Target-induced clustering activates Trim-Away of pathogens and proteins. Nat. Struct. Mol. Biol..

[45] Taxis C., Stier G., Spadaccini R., Knop M. (2009). Efficient protein depletion by genetically controlled deprotection of a dormant N-degron. Mol. Syst. Biol..

[46] Izert M.A., Klimecka M.M., Górna M.W. (2021). Applications of Bacterial Degrons and Degraders—Toward Targeted Protein Degradation in Bacteria. Front. Mol. Biosci..

[47] Bonger K.M., Rakhit R., Payumo A.Y., Chen J.K., Wandless T.J. (2014). General method for regulating protein stability with light. ACS Chem. Biol..

[48] Sun W., Zhang W., Zhang C., Mao M., Zhao Y., Chen X., Yang Y. (2017). Light-induced protein degradation in human-derived cells. Biochem. Biophys. Res. Commun..

[49] Mondal P., Krishnamurthy V.V., Sharum S.R., Haack N., Zhou H., Cheng J., Yang J., Zhang K. (2019). Repurposing Protein Degradation for Optogenetic Modulation of Protein Activities. ACS Synth. Biol..

[50] Pathak G.P., Spiltoir J.I., Höglund C., Polstein L.R., Heine-Koskinen S., Gersbach C.A., Rossi J., Tucker C.L. (2017). Bidirectional approaches for optogenetic regulation of gene expression in mammalian cells using Arabidopsis cryptochrome 2. Nucleic Acids Res..

[51] Longo P.A., Kavran J.M., Kim M.S., Leahy D.J. (2013). Transient mammalian cell transfection with polyethylenimine (PEI). Methods Enzymol..

[52] Piatkevich K.D., Subach F.V., Verkhusha V.V. (2013). Far-red light photoactivatable near-infrared fluorescent proteins engineered from a bacterial phytochrome. Nat. Commun..

[53] Mruk D.D., Cheng C.Y. (2011). Enhanced chemiluminescence (ECL) for routine immunoblotting: An inexpensive alternative to commercially available kits. Spermatogenesis.

[54] Oliinyk O.S., Baloban M., Clark C.L., Carey E., Pletnev S., Nimmerjahn A., Verkhusha V.V. (2022). Single-domain near-infrared protein provides a scaffold for antigen-dependent fluorescent nanobodies. Nat. Methods.

