# Supplementary material for: Photodegradable by Yellow-Orange Light degFusionRed Optogenetic Module with Autocatalytically Formed Chromophore
Source: Int J Mol Sci. 2023 Mar 30;24(7):6526. doi: 10.3390/ijms24076526 (PMC10095432; doi:10.3390/ijms24076526)
Supplement: Supplementary file 1 [file ijms-24-06526-s001.zip › ijms-2285033-supplementary.pdf]

Photodegradable by yellow-orange light degFusionRed optogenetic module with autocatalytically formed chromophore

Konstantin G. Chernov, Kyrylo Yu. Manoilov, Olena S. Oliinyk, Daria M. Shcherbakova, and Vladislav V. Verkhusha

Supplementary Materials

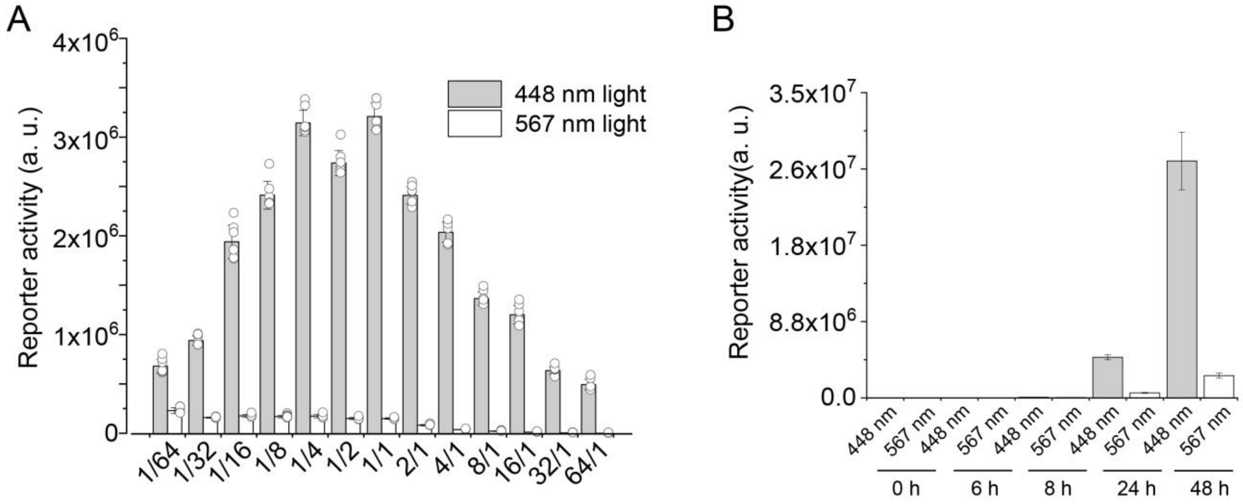

Supplementary Figure S1. Optimization of degFusionRed-based optogenetic transcription system. (**A**) The dependence of luciferase reporter expression on the mass ratio of the transcription factor encoding and reporter encoding plasmids showed in Figure 1A. HEK293 cells were illuminated with 448 nm or 567 nm light for 24 h, and the activity of produced luciferase was determined in a bioluminescence assay. (**B**) Kinetics of luciferase expression under 448 nm and 567 nm illumination from the beginning of transfection to 48 h of the experiment. The mass ratio of the plasmids encoding Gal4-degFusionRed-p65 and luciferase reporter is 4:1.

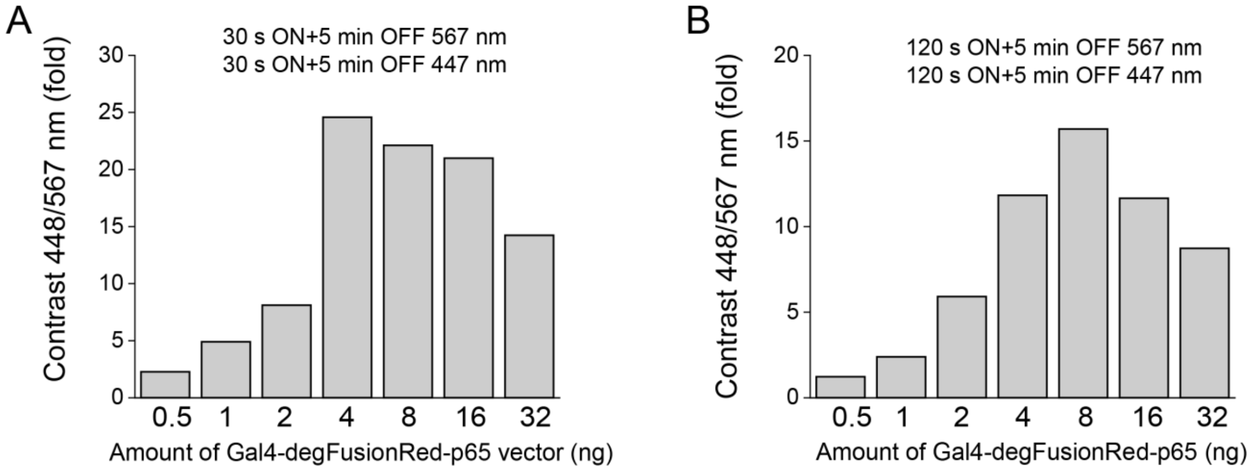

Supplementary Figure S2. Performance of degFusionRed-based optogenetic transcription system under pulsed 448 nm and 567 nm light. (**A**) HEK293 cells were cultured for 24 h under pulsed 448 nm and 567 nm light with a pulse duration of 30 s. (**B**) The pulse duration was increased to 120 s. HEK293 cells were cotransfected with 200 ng of pGL3-Basic vector, 100 ng luciferase reporter plasmid, and 0.5–32 ng plasmid encoding Gal4-degFusionRed-p65 transcription factor (see Figure 1A).

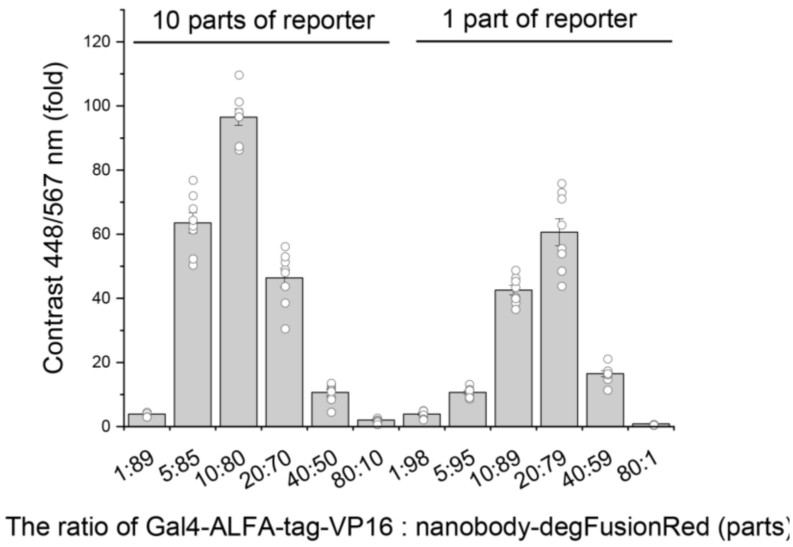

Supplementary Figure S3. Optimization of degFusionRed-based optogenetic system for protein control (Figure 5). Various transfection conditions for the system, where degFusionRed was coupled to Gal4-VP16 via nanobody-ALFA-tag interaction. The ratio of the Gal4-ALFA-tag-VP16 model protein of interest and its targeting anti-ALFA-tag-degFusionRed-fusion was varied from 1:89 to 80:10 in the presence of either 10 parts or 1 part of the luciferase reporter plasmid (Figure 1A bottom). HEK293 cells were cultured for 24 h under pulsed 448 nm or 567 nm light, followed by determining the contrast ratio of the reporter expression.
